# Accelerated exchange of exon segments in Viperid three-finger toxin genes (*Sistrurus catenatus edwardsii*; Desert Massasauga)

**DOI:** 10.1186/1471-2148-8-196

**Published:** 2008-07-08

**Authors:** Robin Doley, Susanta Pahari, Stephen P Mackessy, R Manjunatha Kini

**Affiliations:** 1 Protein Science Laboratory, Department of Biological Sciences, National University of Singapore, 117543, Singapore; 2 CMR Institute of Technology, 132 AECS Layout, IT Park Road, Bangalore 560 037, India; 3 School of Biological Sciences, University of Northern Colorado, Greeley, CO 80639-0017, USA; 4 Department of Biochemistry, Medical College of Virginia, Virginia Commonwealth University, Richmond, VA 23298-0614, USA

## Abstract

**Background:**

Snake venoms consist primarily of proteins and peptides showing a myriad of potent biological activities which have been shaped by both adaptive and neutral selective forces. Venom proteins are encoded by multigene families that have evolved through a process of gene duplication followed by accelerated evolution in the protein coding region.

**Results:**

Here we report five gene structures of three-finger toxins from a viperid snake, *Sistrurus catenatus edwardsii*. These toxin genes are structured similarly to elapid and hydrophiid three-finger toxin genes, with two introns and three exons. Both introns and exons show distinct patterns of segmentation, and the insertion/deletion of segments may define their evolutionary history. The segments in introns, when present, are highly similar to their corresponding segments in other members of the gene family. In contrast, some segments in the exons show high similarity, while others are often distinctly different among corresponding regions of the isoforms.

**Conclusion:**

Ordered, conserved exon structure strongly suggests that segments in corresponding regions in exons have been exchanged with distinctly different ones during the evolution of these genes. Such a "switching" of segments in exons may result in drastically altering the molecular surface topology and charge, and hence the molecular targets of these three-finger toxins. Thus the phenomenon of accelerated segment switch in exons to alter targeting (ASSET) may play an important role in the evolution of three-finger toxins, resulting in a family of toxins with a highly conserved structural fold but widely varying biological activities.

## Background

Snake venom is a mixture of proteins and polypeptides which can be divided into enzymatic and non-enzymatic families [1]. Three-finger toxins are non-enzymatic polypeptides which belong to a well characterized superfamily of snake venom toxins. Structurally they have similar folds, with three β-sheeted loops ('fingers') that are stabilized by 4–5 disulfide bridges present in the central core [2,3]. Despite their structural similarity, they differ widely in their molecular targets. For example, members of this family target various receptor/ion channel proteins such as α1-nAChRs, L-type calcium channels and integrin α_IIb_β_3 _(Table 1). Such a wide diversity in their molecular targets is due to changes in their primary sequences, while keeping the basic molecular scaffold intact. Analysis of amino acid sequences and gene structures will help elucidate the molecular evolution of these functionally important toxins.

**Table 1:** Diversity of molecular targets for representative members of the three-finger toxin gene family.

Toxin	Target	Reference
Short-, long-chain α-neurotoxins	α1-nAChR*	[3]
Long-chain α-neurotoxins	α7 nAChR*	[3]
κ-bungarotoxin	α3 and α4 nAChRs*	[3]
Muscarinic toxins	muscarinic AChRs*	[39]
Calciseptine and FS_2 _toxin	L-type calcium channels	[40,41]
Dendroaspin	α_IIb_β_3 _integrins	[42]
Cardiotoxins	phospholipids and glycosphingolipids	[43,44]
Cardiotoxin A5	α_v_β_3 _integrins	[45]
Fasciculins	acetylcholinesterase	[46]
Hemextin AB complex	blood coagulation factor VIIa	[47]
β-cardiotoxin	β-adrenergic receptors	[48]

*nAChR, nicotinic acetylcholine receptor

As with other snake venom proteins [4-6], three-finger toxins are also encoded by a multigene family [7-10] and contain functionally diversified isoforms. These venom protein families have evolved through a process of gene duplication followed by accelerated point mutations in the protein coding region [11-15]. For example, in phospholipase A_2 _genes, the dN/dS ratio in the coding regions is higher than in the non-coding regions [4,16]. Such adaptive Darwinian evolution plays an important role in the evolution of novel gene functions, leading to functional diversity in each superfamily of toxins. The accelerated rate of mutations in venom proteins likely provides a competitive edge in predator-prey interactions [17].

**Figure 1:**
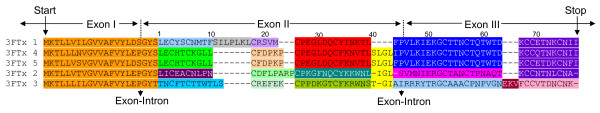
Deduced amino acid sequences of the three-finger toxins. The segments with 85–100% identity are shown in the same colors and less than 65% identity is shown in different colors. The intron-exon boundaries are marked with dotted arrows (exon-intron).

Until recently, three-finger toxins were thought to be present only in venoms of the snake families Elapidae [18]. However, we and others have demonstrated their presence in the venoms of colubrid and viperid snakes [19-22]. Recently we constructed a cDNA library from the venom glands of *Sistrurus catenatus edwardsii *(Desert Massasauga) and identified a three-finger toxin family (0.83% abundance) in the venom gland transcriptome [23]. As three-finger toxins are uncommon in viperid venoms, we performed RT-PCR using a separate pool of RNA as template and found five transcripts that encode three-finger toxins [23]. They have a 21 residue signal peptide followed by a mature protein consisting of 64–68 residues, and the ten conserved cysteine residues, which form five disulfide bridges characteristic of most three-finger toxins, are also present. These viperid toxins belong to the non-conventional three-finger toxin subfamily that has the fifth disulfide bridge in the first loop [24], and they were named 3FTx 1 through 3FTx 5. 3FTx 4 and 5 differ only in one residue in the mature protein, whereas all others are distinct isoforms. All of them have nearly identical signal peptide sequences, but the sequence identity in the mature protein region is often low (31–60%) with the exception of 3FTx 4 and 5 (94% identity) (Figure 1). A systematic comparison of amino acid sequences of the mature proteins indicate that some segments are highly conserved (60–100% identity) between two or three isoforms, whereas other regions are not (only 12.5–50% identity) (see Additional file 1). Such similarities and dissimilarities in various other segments were observed among these three-finger toxins and they appear to be evolving through "switching" of various segments. To understand the molecular evolution of these toxins, we obtained the gene sequences of these three-finger toxins using genomic DNA (gDNA) PCR and GenomeWalking approaches. This is the first report of gene structure of three-finger toxins from snakes of the family Viperidae. The analyses of their gene sequences show that segment "switching" occurs only in the exons, not in the introns. This phenomenon of exchange is likely an important contributor to the gene evolution of this family of toxins which exhibit numerous distinct pharmacological effects.

## Results and Discussion

### Gene structure

**Figure 2:**
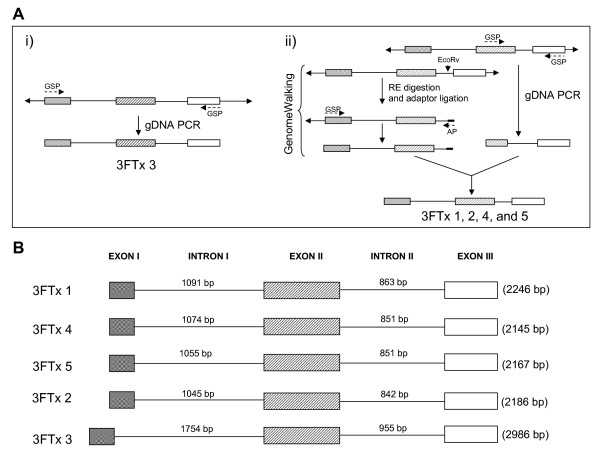
Gene structures of *S. catenatus edwardsii *three-finger toxins. A) Strategy used in determining the gene structures. B) Schematic representation of the gene structures of three-finger toxins. The exon-intron boundaries were determined by comparison with the cDNA sequences. Exons are represented by boxes and introns are represented by solid line. The number of base pairs in the introns and total length are also shown.

**Figure 3:**
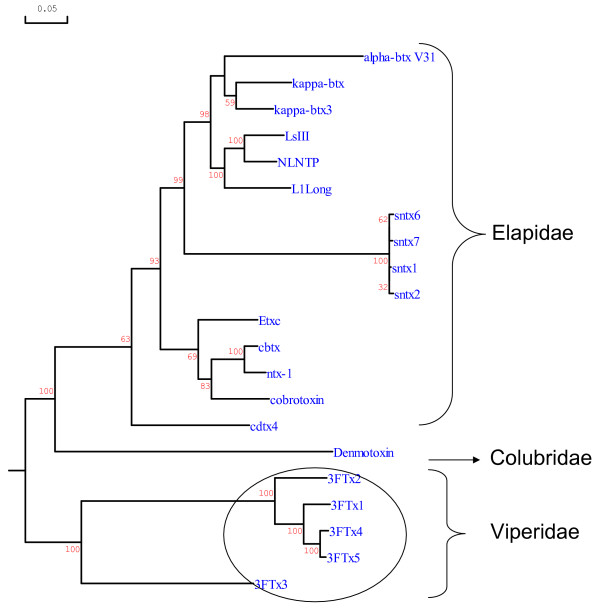
Phylogenetic relationship of representative Viperidae, Colubridae and Elapidae three-finger toxins. Tree was constructed using the software DNAMAN. The gene sequences used were obtained from GenBank and are represented by their name. alpha btx V31 (Y17693), kappa-btx (Y11768) and kappa-btx3 (Y11769) are from *Bungarus multicinctus*; LsIII (AB098531), NLNTP (AB098532), L1Long (AB098533) and Etxc (X51410) are from *Laticauda semifasciata*; sntx1 (AF204969), sntx2 (AF204970), sntx7 (AF204972) and sntx6 (AF204973) are from *Pseudonaja textilis*; cbtx (Y12492), cobrotoxin (Y13399) and cdx4 (Y12493) are from *Naja atra*; ntx-1 (AF096999) is from *Naja sputatrix*; Denmotoxin (EF452300) from *Boiga dendrophila *(Colubridae), and 3FTx 1–5 belong to *S. catenatus edwardsii *(Viperidae).

To understand the evolution of the three-finger toxins in *S. catenatus edwardsii *venom, we determined their gene structures (Figure 2A). We obtained the full length gene of 3FTx 3 by gDNA PCR. However, we obtained only partial genes (from exon II to exon III) of the other four toxins by gDNA PCR even after several attempts, perhaps due to suboptimal annealing of the primers or the thermal cycling profile. We performed GenomeWalking to obtain the remaining gene segments from exon I to exon II (Figure 2A). At least 16 clones were sequenced from each PCR product to obtain the gene sequences of all five three-finger toxins (2.246 kb for 3FTx 1, 2.145 kb for 3FTx 2, 2.186 kb for 3FTx 4, 2.167 kb for 3FTx 5 and 2.986 kb for 3FTx 3). The cDNA sequences were used to determine the exon-intron boundaries (Figure 2B), which follow the GT-AG rule of splice-donor and -acceptor sites [25]. Genes of all five three-finger toxins have similar architecture, with two introns and three exons, similar to those of elapid three-finger toxin genes. Sequence data was deposited in GenBank under accession numbers EU 293789, EU 293790, EU 293791, EU 293792 and EU 293793, respectively. The gene sequences were used to determine the phylogenetic relationship with three-finger toxin genes from the snake families Elapidae and Colubridae. The phylogenetic tree was constructed using DNAMAN, and viperid three-finger toxin genes form a separate cluster in the tree (Figure 3).

#### Intron I

**Figure 4:**
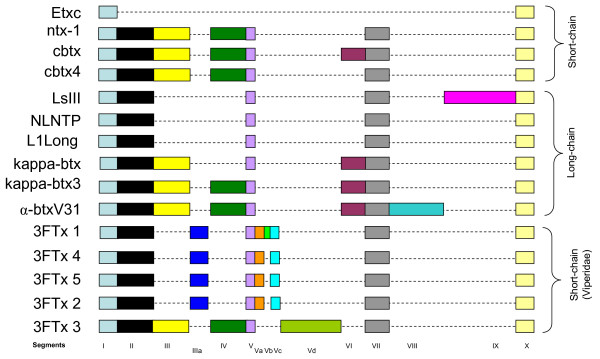
Comparison of intron I structure. The gene sequences were obtained from GenBank and are represented by the gene name (and accession number): Etxc (X51410), ntx1 (AF096999), cbtx (Y12492), cbtx4 (Y12493), LsIII (AB098531), NLNTP (AB098532), L1Long (AB098533), κ-btx (Y11768), κ-btx3 (Y11769) and α-btxV31 (Y17693). Source species are as in Fig. 3. 3FTx 1–5 are gene structures of *S. catenatus edwardsii *three-finger toxins from the present study. The nucleotide sequences were aligned using the online software DIALIGN Multiple Sequence Alignments tool at BiBiServ and then were divided into segments. The nucleotide segments are represented with boxes, and gaps are indicated with a dash. The additional segments in 3FTx 1, 4, 5, 2 and 3 are named IIIa and Va-Vd.

Intron I plays an important role in the expression of various genes [26,27] and is the most variable region in elapid three-finger toxin genes [28]. Sequence alignment of intron I of the viperid genes revealed that it can be divided into segments similar to elapid three-finger toxin genes [28]. Segments I, II, V, VII and X are conserved in all the genes except erabutoxin c gene (Figure 4). In 3FTx 1, 4, 5 and 2, segments III and IV are missing. New additional segments IIIa, Va and Vc are present in 3FTx 1, 4, 5 and 2. 3FTx 1 has an insertion (segment Vb), whereas 3FTx 3 has one additional segment identified as Vd (Figure 4). Interestingly, the additional segments in viperid three-finger gene are short or long nucleotide repeats. Segment Va in 3FTx 1, 2, 4 and 5 has 19–27 continuous "TAA" repeats, while the Vb in 3FTx 1 has 18 continuous "GAT" repeats. These shorter repeats may represent microsatellite sequences. The segment Vd in 3FTx 3 has three different repeats: two repeats of TATTTCATTCCATTCCATATTTTCGATTCTATTCCTGTTCTG (red boxes), three repeats of TCTATTCTATTCCACTCC sequences (blue boxes) and 14 and 27 continuous repeats of CTATT (pink boxes) (see Additional file 2).

Addition/deletion of segments in intron I is also observed in elapid and hydrophiid genes and was linked to the evolutionary diversification of these snake toxin genes [28,29]. Intron I of all viperid three-finger genes are nearly identical; among 3FTx 1, 2, 4 and 5 there is only a short insertion (segment Vb) in 3FTx 1. 3FTx 3 is distinct from other 3FTxs. It has two additional segments (IV and Vd compared to viperid genes) and segment IIIa is missing (Figure 4). The additional segments are either deleted (elapid) or added (viperid) in the gene of three-finger toxins during their evolution, but the role of these insertions/deletions in the expression of the three-finger toxins is currently unknown. Interestingly, the region of exon-intron boundary is similar to elapid and hydrophiid genes and seems to be conserved among all of them.

#### Intron II

**Figure 5:**
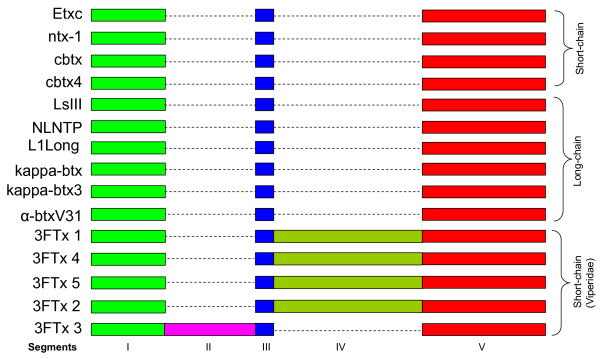
Comparison of intron II structure. The gene sequences were obtained from GenBank (for detail see figure 4). Sequence alignment and division of segments were done as described in figure 4. Segment II is present only in 3FTx 3, and segment IV is present only in viperid three-finger toxin genes (except 3FTx 3).

Intron II of the elapid and hydrophiid three-finger toxin genes is conserved and was thought to be not segmented [28]. However, comparison of the intron II sequences of viperid genes reveals that it also can be divided into segments, similar to intron I (Figure 5). Segments I, III and V are conserved in all three-finger genes. However, 3FTx 1, 4, 5 and 2 genes have an additional common segment (IV), and 3FTx 3 has one additional segments (II). Thus, segmentation appears to be a common structural feature of both introns, and the insertion/deletion of segments may contribute to their regulation of expression. Further, analysis of segments may facilitate understanding the evolutionary history of this unusual gene structural feature.

### Segment switching in exons

**Figure 6:**
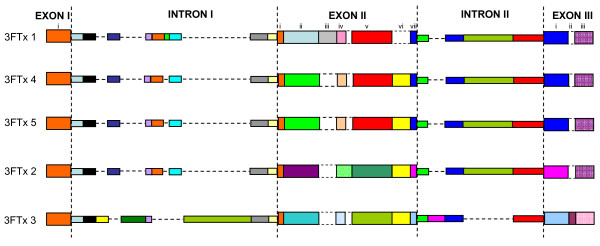
Comparison of gene structures of three-finger toxins from *S. catenatus edwardsii *venom gland transcriptome. Exon-intron boundaries are marked by vertical dashed lines and similar sequences are represented by segments. Exons are divided into sub segments on the basis of their similarities (same color) and dissimilarities (different color). Absence of a corresponding segment in the exons is shown by a dashed line box. The segments in the introns are conserved (same color), and absence of a corresponding segment is represented by a dashed line.

Exons also appeared to have segments (Figure 1). As expected because of overall size, the segments in exons are much smaller as compared to those in introns (4–7 bp in segment vii in exon II to 53 bp in segment i in exon III, compared to 21–22 bp in segment X to 680 bp in segment Vd in intron 1). Analysis of the gene sequences reveals that the gain/loss of segments occurs in both introns and exons (Figure 6). The segments in introns, when present, show high similarities (>85% identity). In contrast, while some exon segments show high sequence identity (60–100% identity; shown in same colors), other segments show low identity (12.5–50% identity; shown in different colors). Such similarity/dissimilarity of segments is more prominent in exon II and exon III. In exon II, there are four different kinds of segment ii; in 3FTx 4 and 5 they are identical, whereas in the other genes they are totally different (Figure 6). In the same way, segment v is similar in 3FTx 1, 4 and 5, whereas in 3FTx 2 and 3 it is different. Segment vi is absent in 3FTx 1 but present in the other genes; this segment is similar in 3FTx 4 and 5 but different in 3FTx 2 and 3. Thus exon II appears to have evolved through "switching" of segments, although the origins of the "new" segments are not known. Similarly, exon III also appears to have evolved through "switching" of segments. However, this phenomenon is not observed in introns of these genes, although there are additions/deletions of a few short segments in introns (see Additional file 1). The switching of segments in exons has far reaching effects on the biological effects of the toxins. The changed segments will affect the overall surface properties of the expressed protein (such as charge density and hydrophobicity) and hence the function. In contrast, the small additions/deletions in introns will not affect the nature of the protein product, but may affect its expression. It is important to note that in spite of this segment switching, positions of cysteine residues are conserved, thereby maintaining similar disulfide pairing, folding architecture and the overall three-finger fold of the mature protein.

There are several possibilities that could explain the observed switching of segments.

1. Splicing variations: The difference in isoforms of some proteins due to change of segments can be easily explained based on splicing variation [30]. However, unlike these proteins, the segment switching in viperid 3FTx occurs within exon II and exon III. Among three-finger toxins, long-chain neurotoxins arose from short-chain neurotoxins through an error in the splicing site [31]. Such an error leads to insertion of a short segment with the fifth disulfide in the second loop. In viperid toxin genes, however, the insertions of segments do not occur at the intron-exon boundaries, so the mechanism of insertions/deletions or switching of segments does not occur due to errors in splicing.

2. Recombination: Distinct genes encoding isoforms of proteins are also generated through recombination of two related/unrelated genes [32,33]. In general, the segments involved in such recombination events are fairly large (700 to 2500 bp), and the segments that are exchanged in exons of 3FTxs are probably much too small. Therefore, canonical recombination may not be involved in these exchange events.

3. Accumulation of point mutations: Accelerated point mutations in three-finger toxins are common and they lead to the evolution of several isoforms [10,11,14]. Although these point mutations occur in exon segments, they may not explain such a distinct change in the sequences of segments. This possibility requires the repeated occurrence and accumulation of multiple point mutations within each segment. Further, all the intermediates have to be selected through evolution. The absence of intermediate isoforms, however, contradicts this possibility; and

4. Independent recruitment events: Venom protein genes are thought to be recruited to the venom gland genome by gene duplication of a normal physiologically important gene and recruitment of the duplicated gene for expression in the venom gland [34]. It is possible, but not probable, that each of these isoforms has an independent origin and their ancestral three-finger toxin genes were recruited at different times. High similarity across numerous 3FTx genes in exon I, and introns I and II, supports instead a single recruitment and lineage of these genes and not multiple recruitment events. In the unlikely events of independent recruitments, introns will have to undergo convergent evolution to explain the high similarity while the exons will be undergoing divergent evolution. Therefore, segment switching results in divergence of functional regions of exons (see below) while maintaining the basic fold, rather than convergence upon a single scaffold motif during independent recruitment.

Although the mechanism of the exchange of segments in exons is unknown, it is apparent that these events play important roles in the evolution of these toxins, in addition to the role that accelerated point mutations in the exons plays in toxin evolution [10,11,14]. These point mutations appear to alter the interaction surfaces of toxins [35]. However, they affect one residue at a time and a smaller molecular surface, and they may help in fine-tuning of functional sites of the molecule for interaction with specific molecular targets. They may (a) enhance the affinity to a specific receptor or ion channel; (b) change the specificity to another closely related receptor or ion channel; and (c) change the species specificity of the toxin to a particular receptor. In contrast, accelerated exchange of larger segments drastically changes parts of the interacting loops or toxin surface. Therefore, these exchanges of segments may help in switching the molecular targets of toxins and hence affecting their pharmacological properties. We propose that accelerated segment switch in exons to alter targeting (ASSET) is a phenomenon which plays an important role in "remodeling" a toxin toward a different and novel receptor target (see below).

### ASSET and evolution of molecular surfaces

**Figure 7:**
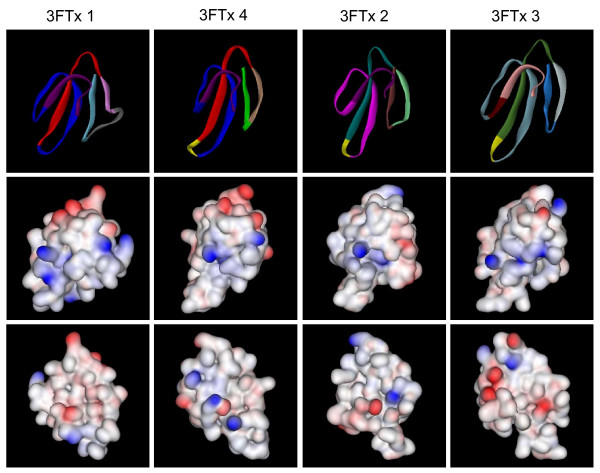
Three-dimensional models of three-finger toxins from *S. catenatus edwardsii *venom gland transcriptome. Top row shows the solid ribbon models. Segments are color coded as in Figure 1. Middle and bottom (180° rotation) rows show the electrostatic potential of both the surface. The positively and negatively charged residues are shown in blue and red colors, respectively, and the hydrophobic residues are shown in white color.

To understand the impact of switching of segments on the molecular surfaces of three-finger toxins in *S. catenatus edwardsii*, we modeled all four distinct three-finger toxins. As shown in Figure 7, the three β-sheeted loops in these toxins are distinctly different from one another, as most of them are replaced by segment switching (Figure 7, top row). Further, the electrostatic potentials of these toxins indicate that the charge distributions on their molecular surfaces are also different. 3FTx 1 and 4 have more acidic residues on the surface as compared to 3FTx 2 and 3 (Figure 7, middle and bottom rows). This drastic difference in the charge residues on the surface is due to the exchange of segments (see Additional file 1), but retention of the similar molecular fold. Such a change in the charged residues might play an important role in switching the molecular targets. Since most of the functional sites are located on these β-sheeted loops (for a review see, [36]), it is logical to propose that all of these novel viperid toxins have distinct pharmacological properties. Therefore, ASSET phenomena affect the molecular surfaces of three-finger toxins significantly and alter their molecular targets, playing a crucial role in the evolution of the three-finger toxins.

## Conclusion

Systematic analyses of gene sequences of *Sistrurus catenatus edwardsii *(Desert Massasauga) three-finger toxins indicate that short segments in exons II and III are changed more rapidly compared to intron segments. We propose that such a phenomenon (ASSET) of accelerated segment switching in exons has the effect of rapidly altering the molecular surface properties. This mechanism of rapid change can provide a selective advantage to venomous snakes in predator/prey coevolutionary arms races, resulting in a diversity of structurally similar toxins in a single venom and allowing the venom toxins repertoire to stay a step ahead of prey defensive responses [1,17]. Thus ASSET plays an important role in changing the molecular target and hence the pharmacology of these toxins.

## Methods

### Tissues and reagents

Extraction of venom glands and liver from *Sistrurus catenatus edwardsii *(Desert Massasauga) were reported previously (Pahari et al. 2007). RNeasy mini kit, DNeasy DNA extraction kit, Qiaquick gel extraction and PCR purification kit, and a QiaPrep mini prep kit were purchased from Qiagen (Valencia, CA, USA). GenomeWalker™ kit was purchased from Clontech Laboratories Inc (Palo Alto, CA, USA), Long PCR Enzyme Mix was procured from Fermentas (Burlington, ON, Canada), TOPO^® ^XL PCR Cloning kit was obtained from Invitrogen (Carlsbad, CA, USA), and ABI PRISM^® ^BigDye^® ^terminator cycle sequencing ready reaction kit was purchased from Perkin Elmer (Foster City, CA, USA). Oligonucleotides were custom synthesized by 1^st ^BASE (Singapore). All other chemicals and reagents used were of the purest grade available.

### Genomic DNA extraction

Genomic DNA (gDNA) was extracted from the *S. catenatus edwardsii *liver tissue (30 mg, previously stored in RNAlater) using the DNeasy Tissue kit (Qiagen, USA) according to the manufacturer's instructions. RNaseH was used during the extraction to remove contaminating RNA. The integrity of the isolated gDNA was confirmed by 0.8% agarose gel electrophoresis and DNA was quantified spectrophotometrically.

### gDNA PCR

gDNA PCR was performed to obtained the gene sequence of 3FTx 3 using a gene-specific forward primer 5'-ATGAAAACTCTGCTGTTGATCCTGGGGGT-3' and gene-specific reverse primer 5'-GCCAATAGTCACTTTTAGAACTATTTGTTGCAGTTGTCTG-3'. The PCR reaction contained 1.0 unit of Long PCR Enzyme Mix, 1 μg of gDNA. 0.2 mM dNTP, 0.2 μM primers and 1 × Long enzyme mix polymerase buffer in a total of 50 μL. The thermal cycling reaction involved 35 cycles of one step each at 95°C for 15 s, 60°C for 15 s, 68°C for 3 min followed by a final extension step at 68°C for 10 min. The amplified PCR products were extracted and cloned as mentioned below.

### Construction of GenomeWalking libraries

The GenomeWalking libraries were constructed using the Universal GenomeWalker™ kit (Clontech Laboratories Inc, USA) according to the manufacturer's instructions. Briefly, libraries were constructed with 3 μg of gDNA restriction digested with DraI, EcoRV, PvuII and StuI. The 'genome walk' involved two sets of primers: adaptor primer 1 (AP1-sense) 5'-GTAATACGACTCACTATAGGGC-3' and nested PCR adaptor primer 2 (AP2-sense) 5'-ACTATAGGGCACGCGTGGT-3', both provided in the kit, and 25-mer and 27-mer gene-specific primers designed from the signal peptide regions of cDNAs of all the three-finger toxins. Primary and nested PCRs were performed as recommended by the manufacturer (BD GenomeWalker™) using Advantage Polymerase 2 Mix obtained from Clontech Laboratories Inc (Palo Alto, CA, USA). The 50.0 μl reaction mixture consisted of 1 μl of DNA template (0.1 μg) (either from each library or from primary PCR products), 1× PCR buffer (provided in the kit), 0.2 mM dNTPs, 0.2 μM appropriate adaptor primers, 0.2 μM of appropriate gene-specific primers, 1× Advantage™ 2 polymerase mix. The thermal cycling profile used was as follows: 7 cycles of 94°C for 2 s, 72°C for 3 min; 32 cycles of 94°C for 2 s, and 67°C for 4 min followed by a final extension at 67°C for 7 min. The PCR products were purified, cloned and sequenced.

### Cloning and sequencing

PCR products were subjected to 1% agarose gel electrophoresis, visualized by ethidium bromide staining and purified using a gel extraction kit or PCR purification kit. Purified PCR products were ligated either to pDrive vector (Qiagen, Hilden, Germany) or pCR^®^-XL-TOPO^® ^vector (Invitrogen, USA). Ligated vectors were transformed to DH5a competent cells by heat shock. Kanamycin (100 mg mL^-1^) was used for antibiotic resistance selection. Blue/white colony screening was done on LB agar plates using 80 mg mL^-1 ^X-gal and 0.5 mL L^-1 ^of 100 mM isopropyl-β-D-thiogalacto-pyranoside (IPTG) to select the positive colonies.

DNA sequencing reactions were carried out using the ABI PRISM^® ^BigDye^® ^terminator cycle sequencing ready reaction kit (BDV3.1) according to manufacturer's instructions (Applied Biosystem, Foster City, CA, USA). DNA sequencing was carried out using an ABI PRISM^® ^3100 automated DNA sequencer.

### Sequence analysis and phylogenetic tree

Sequence analysis was carried out using the BLASTX program at National Center for Biotechnology Information website. Multiple sequence alignment was done using DNAMAN and online DIALIGN Multiple Sequence Alignments tool at BiBiServ [37]. A neighbor-joining tree was constructed using DNAMAN version 4.1.5.1 (Lynnon BioSoft).

### Molecular modeling

Three dimensional structures of three-finger toxins from Desert Massasauga (*Sistrurus catenatus edwardsii*) venom gland transcriptome were modeled using the online I-TASSER server for protein 3D structure prediction [38]. The server predicts the folds and secondary structure by profile-profile alignment (PPA) threading techniques. For each protein, 3–4 models were obtained. The model with the lowest free energy was used for further analysis. Ribbon structure diagrams and surface charge models were created using the DS ViewerPro software to compare potential differences in electrostatic charges of these viperid 3FTx.

## Abbreviations

ASSET, Accelerated Segment Switch in Exons to alter Targeting

## Authors' contributions

RD and SP conducted the wet lab experiments to determine the gene structure of the three-finger toxins. RD created the figures, tables and wrote the manuscript; SPM supplied the liver and venom glands and significantly contributed to the manuscript writing; RMK contributed to the data analyses and writing of the manuscript and also supervised RD and SP All authors contributed to the development of the concept.

## Supplementary Material

Additional File 1 Identity between the exon segments of the three-finger toxins. The gene sequence of the three-finger gene was divided into various segments and percent identity between these segments is shown in the table. Percent identity above 50% is shaded with grey color. Click here for file

Additional File 2 Comparison of gene structure of *Sistrurus catenatus edwardsii *three-finger toxin genes. The gene sequence of the three-finger gene was divided into various segments and similar/dissimilar color coding was given on the basis of sequence homology. Click here for file
