# Supplementary material for: Accelerated exchange of exon segments in Viperid three-finger toxin genes (Sistrurus catenatus edwardsii; Desert Massasauga)
Source: BMC Evol Biol. 2008 Jul 8;8:196. doi: 10.1186/1471-2148-8-196 (PMC2474615; doi:10.1186/1471-2148-8-196)
Supplement: Additional File 1 — Identity between the exon segments of the three-finger toxins. The gene sequence of the three-finger gene was divided into various segments and percent identity between these segments is shown in the table. Percent identity above 50% is shaded with grey color. [file 1471-2148-8-196-S1.pdf]

Additional file 1: Identity between the amino acid residues in exon segments of the three-finger toxins. Numbers in bracket of the segments are as in figure 1.

A

3FTxs	% identity with 3FTx1							
	Segment 1 (1-10)	Segment 2 (11-17)	Segment 3 (18-25)	Segment 4 (26-39)	Segment 5 (40-44)	Segment 6 (45-64)	Segment 7 (65-67)	Segment 8 (68-78)
3FTx 4	40.00	Nil	16.67	78.57	Nil	95.00	Nil	90.91
3FTx 5	40.00	Nil	16.00	78.57	Nil	95.00	Nil	81.82
3FTx 2	40.00	Nil	12.50	50.00	Nil	45.00	Nil	63.64
3FTx 3	16.00	Nil	33.3	21.43	Nil	15.00	Nil	45.45

B

3FTxs	% identity with 3FTx4							
	Segment 1 (1-10)	Segment 2 (11-17)	Segment 3 (18-25)	Segment 4 (26-39)	Segment 5 (40-44)	Segment 6 (45-64)	Segment 7 (65-67)	Segment 8 (68-78)
3FTx 1	40.00	Nil	16.67	78.57	Nil	95.00	Nil	63.64
3FTx 5	100.00	Nil	100.00	100.00	100.00	95.00	Nil	72.73
3FTx 2	30.00	Nil	16.00	50.00	60.00	45.00	Nil	63.64
3FTx 3	25.00	Nil	16.67	35.71	60.00	15.00	Nil	45.45

C

3FTxs	% identity with 3FTx2							
	Segment 1 (1-10)	Segment 2 (11-17)	Segment 3 (18-25)	Segment 4 (26-39)	Segment 5 (40-44)	Segment 6 (45-64)	Segment 7 (65-67)	Segment 8 (68-78)
3FTx 1	40.00	Nil	12.50	50.00	Nil	45.00	Nil	63.64
3FTx 4	30.00	Nil	12.50	50.00	60.00	45.00	Nil	63.64
3FTx 5	30.00	Nil	12.50	50.00	60.00	45.00	Nil	54.55
3FTx 3	16.67	Nil	12.50	40.00	60.00	25.00	Nil	50.00

D

3FTxs	% identity with 3FTx3							
	Segment 1 (1-10)	Segment 2 (11-17)	Segment 3 (18-25)	Segment 4 (26-39)	Segment 5 (40-44)	Segment 6 (45-64)	Segment 7 (65-67)	Segment 8 (68-78)
3FTx 1	16.67	Nil	33.33	21.43	Nil	15.00	Nil	45.45
3FTx4	25.00	Nil	16.67	35.71	60.00	15.00	Nil	45.45
3FTx5	25.00	Nil	16.67	35.71	60.00	15.00	Nil	45.45
3FTx 2	16.67	Nil	12.50	40.00	60.00	25.00	Nil	50.00

The segments above 60% identity are shown in grey boxes.
