# Supplementary material for: Accelerated exchange of exon segments in Viperid three-finger toxin genes (Sistrurus catenatus edwardsii; Desert Massasauga)
Source: BMC Evol Biol. 2008 Jul 8;8:196. doi: 10.1186/1471-2148-8-196 (PMC2474615; doi:10.1186/1471-2148-8-196)
Supplement: Additional File 2 — Comparison of gene structure of Sistrurus catenatus edwardsii three-finger toxin genes. The gene sequence of the three-finger gene was divided into various segments and similar/dissimilar color coding was given on the basis of sequence homology. [file 1471-2148-8-196-S2.pdf]

Additional file 2. Comparison of gene structure of *Sistrurus catenatus edwardsii* three-finger toxin genes. Similar color coding is given to segments in introns on the basis of sequence homology with other three-finger gene. Exons are marked by double headed arrows; the intron-exon boundary is marked by thick red color arrows. The segments in exons are numbered as in Figure 5. The pink, blue and red boxes are repeats in intron segment Vd as in Figure 5.

```
                                     Exon I
                 <------------------------------------------------->  ↓
                                        i                                                                                          I
3FTx1   ATGAAAACTCTGCTGGTGATCTTGGGGGTGGTGGCATTCGTGTACCTGGACTCAG-GTAAGGCTGTCAAGCTTTGATCTTGGGTGACAATCAAACAAAAAAAAGG--GGTGTA---GCGGGAGGTATGTTATTGGAGGCAGTTGT
3FTx4   ATGAAAACTCTGCTGTTGAACGTGGGGGTGGTGGCATTCGTGTACCTGGAGCCAGGGTAAGGTTGTCAGGCTTTGATCTTGGGTGACAATCAGACAAAAAAAAGGAGGGTGCA---GAGGGAGGTATGGTATTGGAGGCAGTTGT
3FTx5   ATGAAAACTCTGCTGGTGAGCGTGGGGGTGGTGGCATTCGTGTACCTGGACTCAG-GTAAGGCTGTCAAGCTTTGATCTTGGGTGACAATCAAACAAA-AAAAGG--GGTGTA---GCGGGAGGTATGTTATTGGAGGCAGTTGT
3FTx2   ATGAAAACTCTGCTGGTGACCGTGGGGGTGGTGGCATTCGTGTACCTGGAGCCAG-GTAAGGCTGTCAAGCTTTGATCTTGGGTGACAATCAAACAAAAAAAAGG--GGTGTA---GCGGGAGGTATGTTATTGGAGGCAGTTGT
3FTx3   ATGAAAACTCTGCTGTTGATCCTGGGGGTGGTGGCATTCGTGTACCTGGAGCCAG-GTAAGGTTGTCAAGCTTTGATCTTGGGTGACAATTAAACCAAAAAC-AAAGGGACAGAGGGAGGTACG-AT------------AGTTGT

                                              I                                                                       II
3FTx1   GGTCCATTTTGTTGGTGGGGTTTT----CTCTTTTGCTCTGCATCAGGAGGGTCCATGGGAGCCTTGAGGGGAGAGGACTTA-CTCTCCGTTTGGAGAGAAGCTCATAGCAGAGACAAAGGCTCTTCCAGGCTGGCATTCTGGGG
3FTx4   GGTCCATTTTGTTGGTGGGGTTTT----CTCTTTTGCTCTGCATCAGGAGGGTCCATGGGAGCCTTGAGGGGAGAGGAGTTG-CTCTCCGTTTAGAGAGAAGCTCATAGCAGAGACAGAGGCTCTTCCAGGCTGGCATTCTGGGG
3FTx5   GGTCCATTTTGTTGGTGGGGTTTT----CTCTTTTGCTCTGCATCAGGAGGGTCCATGGGAGCCTTGAGGGGAGAGGACTTA-CTCTCCGTTTGGAGAGAAGCTCATAGCAGAGACAAAGGCTCTTCCAGGCTGGCATTCTGGGG
3FTx2   GGTCCATTTTGTTGGTGGGGTTTT----CTCTTTTGCTCTGCATCAGGAGGGTCCATGGGAGCCTTGAGGGGAGAGGACTTA-CTCTCCGTTTGGAGAGAAGCTCATAGCAGAGACAAAGGCTCTTCCAGGCTGGCATTCTGGGG
3FTx3   GGTCCACTCTGTTGTTGGGTCTTTCTCACTCTTTTGCTCTGCATCAGGAGGGTCCATGG-AGCCTTCAGGGTAGAAAGTGGAATTCTCCATTTGGAAAGAAGCCCATA------------GCTGTTCCAGGCTGGCCCTCTGGGA

                                                                         II
3FTx1   TAGACTGCTTCTGAAAGGAGAGGCTCCTTTGGGTAGTCAATTCTGCCCTTAGA-ATTACTCCTGGGAGTGGGATGAAAAATCTCCCCTTTTCTTAATCCTCCATCTTGTCTATTTCTGGC------------------TTTGGAT
3FTx4   TAGACTGCCTCTGAAAGGAGAGGCTCCTTTGGGTAGTCAATTCTGCCCTTAGA-ATTACTCCTGGGAGTGGGATGAAAAATCTCCCCTTTTCTTAGTCCTCCATCTTCTCTATTTCTGGCTTGGATGGTATTTCAGTATTTGGAT
3FTx5   TAGACTGCTTCTGAAAGGAGAGGCTCCTTTGGGTAGTCAATTCTGCCCTTAGA-ATTACTCCTGGGAGTGGGATGAAAAATCTCCCCTTTTCTTAATCCTCCATCTTGTCTATTTCTGGC------------------TTTGGAT
3FTx2   TAGACTGCTTCTGAAAGGAGAGGCTCCTTTGGGTAGTCAATTCTGCCCTTAGA-ATTACTCCTGGGAGTGGGATGAAAAATCTCCCCTTTTCTTAATCCTCCATCTTGTCTATTTCTGGC------------------TTTGGAT
3FTx3   TAGACTGCCTCTGAAAGGAGAGGTTCCTTTGTGTAGTGAATTCTGCC--ATGAAATGAATCCTGGCAATGAAGTGAAAAATATCTCCTTTTCTTAGCCATCCGTTCCGCCTCTCTCTGGCTTTGCCACCATTCAGTATTTCGGAG

                         II                                                                 III                                                   IIIa
3FTx1   GGCGCAGCCAAAGGGGATTAGTGATGCTTCCTTCAAGTCAAGA-------------------------------------------------------------------------------------TTATGCACAATTTTGGA
3FTx4   GGCGCAGCCAAAGGGGATCAGGGCTGCTTCCTTCAAGTCAAGA-------------------------------------------------------------------------------------TTATGCACAATTTTGGA
3FTx5   GGCGCAGCCAAAGGGGATTAGTGATGCTTCCTTCAAGTCAAGA-------------------------------------------------------------------------------------TTATGCACAATTTTGGA
3FTx2   GGCGCAGCCAAAGGGGATTAGTGATGCTTCCTTCAAGTCAAGA-------------------------------------------------------------------------------------TTATGCACAATTTTGGA
3FTx3   GATACGGCCAAAAGGGAGTGATCATGCCTCCTTCAAGTCAAATCTGCACATTCCTCAACATCAGCCTGAGATTGCTGGGATTTCCTAGAATCCTCAATCTATCATTCAACTC---TGGGGGTCTCCCA

                                     IIIa                                                                              IV
3FTx1   AAAAAAATGAAAAAAAAAAAAAAACCCCTACAGAGGAGAATATAATTGAAAAAA-TACTGGATAGGTTAATGTTAAAAATTA----------------------------------------------------------------
3FTx4   AAAAAAATGAAAAAAAAAAAAA-CCC--ACAGAGGAGAATATGATTGAAAAAAATACTGGATAAGTTAATGTTAAAAATTA----------------------------------------------------------------
3FTx5   AAAAAAATGAAAAAAAAAAAAA-CCCCTACAGAGGAGAATATAATTGAAAAAAATACTGGATAGGTTAATGTTAAAAATTA----------------------------------------------------------------
3FTx2   AAAAAAATGAAAAAAAAAA----CCCCTACAGAGGAGAATATAATTGAAAAAAATACTGGATAGGTTAATGTTAAAAATTA----------------------------------------------------------------
3FTx3   ---------------------------------------------------------------------------------TCCCAGGATAAACCAACCCTAACCTGCTTAGCTTCCAGACAGAGACCAAACACTGAAGTTTCCA

                                                                         IV
3FTx1   -------------------------------------------------------------------------------------------------------------------------------------------------
3FTx4   -------------------------------------------------------------------------------------------------------------------------------------------------
3FTx5   -------------------------------------------------------------------------------------------------------------------------------------------------
3FTx2   -------------------------------------------------------------------------------------------------------------------------------------------------
3FTx3   TTTGCTTATATTTTATGGCCTGTTTCAGAAGACGTGATTTTTTTTTTTAAATCCCAGGTTTTATCCCAGGCATGGTGGCACAGTGGTTAGAGTATTTCAGGCGAATTCTGCCGAATGCCAACTGCCAGCAGTTTGGCAGTTCGAT
```

```
                                                                     IV
3FTx1   ------------------------------------------------------------------------------------------------------------------------------------------------
3FTx4   ------------------------------------------------------------------------------------------------------------------------------------------------
3FTx5   ------------------------------------------------------------------------------------------------------------------------------------------------
3FTx2   ------------------------------------------------------------------------------------------------------------------------------------------------
3FTx3   CCTTACTGGGTCAAGATTGACTCAGCCTTCCATCCTTTCTGGGGCTGGTAAACTGAGGACCTAAATTGTTGAGCATAATATGCTGACTCTGTAAACTGCTTAGAAAGGGCTGTAAAATCCTGTGAAGAGGTATAAAAGTCTCA

                   IV                       V                                                Va
3FTx1   --------------------------AAGAAAAAA-AAGAAACTGAATTCTATATGACGTGGGGTCTTTGATCGTTACACAATTGATGTTAATGGGGGAAATACTATTTTTAACAATGTGATTGCTATTTCCTAGTATATGATTG
3FTx4   --------------------------AAGAAAAAG-AAGAAACTGAATTCTATATGACATGGGGTCTTTGATTGTTATACAATTAATGTTAATGGGGGAAATGCTATTTTTAACAATGTGATTGCTATTTCCTAGTATATGATTG
3FTx5   --------------------------AAGAAAAAA-AAGAAACTGAATTCTATATGACGTGGGGTCTTTGATCGTTACACAATTAATGTTAATGGGGGAAATACTATTTTTAACAATGTGATTGCTATTTCCTAGTATATGATTG
3FTx2   --------------------------AAGAAAAAAAAAGAAACTGAATTCTATATGACGTGGGGTCTTTGATCGTTACACAATTAATGTTAATGGGGGAAATACTATTTTTAACAATGTGATTGCTATTTCCTAGTATATGATTG
3FTx3   GTGCTATTGCTATTTAGTAATCACTGAAGGGAAAG-AAGAAACCTATTACTTCTTCCTCCTATTT--------------------------------------------------------------------------------

                                                                        Va
3FTx1   TATTTTGATAAGATTATTATGAATGTGATGATTATTATATTCTAATACTTGTATACCTAGACAACGCACTGTTCAATACCGAAATGGAATTTTTGAGGTAATAATAATAATAATAATAATAATAATAATAATAATAATAATAATAATA
3FTx4   TATTTTGATAAGATTATTATGAATGTGATGATTATTATATTCTAATACTTGTATACCTAGACAACGCACTGTTCAATACCGAAATGGAATTTTTGAGGTAATAATAATAATAATAATAATAATAATAATAATAATAATAATAATAATA
3FTx5   TATTTTGATAAGATTATTATGAATGTGATGATTATTATATTCTAATACTTGTATACCTAGACAACGCACTGTTCAATACCGAAATGGAATTTTTGAGGTAATAATAATAATAATAATAATAATAATAATAATAATAATAATAATAATA
3FTx2   TATTTTGATAAGATTATTATGAATGTGATGATTATTATATTCTAATACTTGTATACCTAGACAACGCACTGTTCAATACCGAAATGGAATTTTTGAGGTAATAATAATAATAATAATAATAATAATAATAATAATAATAATAATAATA
3FTx3   -------------------------------------------------------------------------------------------------------------------------------------------------

                  Va                                                Vb                                                 Vc
3FTx1   ATAATAATAAT-------------------------GATGATGATGATGATGATGATGATGATGATGATCATGATGATGATGATGATGATGATGAGGTTCCCTTTTCTTAGCCATCTCTCCTAGTTTCTAGAGCATTTGATCTTC
3FTx4   ATAATAATAATAATAATAATAATAATAATAATAAAT------------------------------------------------------------GTTCCCTTTTCTTAGCCGTCTCTCCTAGTTTCTAGAGCATTTGATCTTC
3FTx5   ATAATAATAATAATAATAATAATAATAATAATAAAT------------------------------------------------------------GTTCCCTTTTCTTAGCCGTCTCTCCTAGTTTCTAGAGCATTTGATCTTC
3FTx2   ATAATAATAATAATAATAATAATAAAT---------------------------------------------------------------------GTTCCCTTTTCTTAGCCGTCTCTCCTAGTTTCTAGAGCATTTGATCTTC
3FTx3   -------------------------------------------------------------------------------------------------------------------------------------------------

         Vc                                                                    Vd
3FTx1   ATAT---------------------------------------------------------------------------------------------------------------------------------------------
3FTx4   GCAT---------------------------------------------------------------------------------------------------------------------------------------------
3FTx5   GCAT---------------------------------------------------------------------------------------------------------------------------------------------
3FTx2   GCAT---------------------------------------------------------------------------------------------------------------------------------------------
3FTx3   ----TTTCCTAGAGAGGGAGAGAGAAAGAACGTGCCTAAGTGAGCTTTGATGCCTAAATTGGGACTAATTTCTAGACCATTTGATCTCGGTTCTCCTGCTTCAGTGGGGGGGGGGGCTTAGAAGACCTCCAGGGTGCCTTTCAGC

                                                                        Vd
3FTx1   -------------------------------------------------------------------------------------------------------------------------------------------------
3FTx4   -------------------------------------------------------------------------------------------------------------------------------------------------
3FTx5   -------------------------------------------------------------------------------------------------------------------------------------------------
3FTx2   -------------------------------------------------------------------------------------------------------------------------------------------------
3FTx3   TCTATTCTATTCTATTCTATTCTATTCTATTCTATTCTATTCTATTCTATTCTATTCTATTCTATTCTATTCTATTCCACTCCTATTCTATTCTATTTCATTCCATTCCATATTTTCGATTCTATTCCTGTTCTGTTCTATTCTATTCCA

                                                                         Vd
3FTx1   -------------------------------------------------------------------------------------------------------------------------------------------------
3FTx4   -------------------------------------------------------------------------------------------------------------------------------------------------
3FTx5   -------------------------------------------------------------------------------------------------------------------------------------------------
3FTx2   -------------------------------------------------------------------------------------------------------------------------------------------------
3FTx3   CTCCATTCCACTCCACTCCATTTTATTTCATTCCATTCCATATTTTCGATTCTATTCCTGTTCTGTTCCATTCCATATTTTCTATTCTATTCCATTCCATTCCATATTTTCTATTCTATTCTATTCCACTCCATTCTATTCTATT
```

```
                                                                              Vd
3FTx1     -------------------------------------------------------------------------------------------------------------------------------------------------
3FTx4     -------------------------------------------------------------------------------------------------------------------------------------------------
3FTx5     -------------------------------------------------------------------------------------------------------------------------------------------------
3FTx2     -------------------------------------------------------------------------------------------------------------------------------------------------
3FTx3     CCATTCCATTCCACTCCACTCCACTCCATTCCATTCCATATTTTCTATTCTATACCTGTTCTGTGTTCTATTCTATTATATTCTATTCTACTCTACTCTATTCTATTCTATTCTATTCTATTCTTTTCTATTCTATTCTATTCTA

                                                Vd                                                               VII
3FTx1     --------------------------------------------------------------------------------------------------------GGATATTTTTACTGCTGGCTTTGCTATGAGGTTATCTGTTG
3FTx4     --------------------------------------------------------------------------------------------------------GGATATTTTCACTGCTGGCTTTGCTATTAAATGATCTATCA
3FTx5     --------------------------------------------------------------------------------------------------------GGATATTTTCACTGCTGGCTTTGCTATTAAATGATCTATCA
3FTx2     --------------------------------------------------------------------------------------------------------GGATATTTTCACTGCTGGCTTTGCTATTAAATGATCTATCA
3FTx3     TTCTATTCTATTCTATTCTATTCTATTCTATTCTATTCTATTCTATTCTATTCTATTGCATATTTTCTATTCTATTCTATCCTATTCTATTCTAATCTTTCTAGGGAAATTTTCACAGCTGATCTTTCTATTAAACAATCTATCA
                                                                                                          Exon II
                                                                                  <--------------------------------------------------------------->
                          VII                                   X                   i          ii                           iii         iv
3FTx1     TCTAATACATAATAAGATGGCAAGATCAGAAAAATAAATATCCAACTAAGGTTTCTATGTTTTATTTT-GCTCATCTAGGATACTCCCTGGAATGTTATTCATGCAATATGACTTTTTCTATTCTACCTCTAAAGCTGTGTAGGT
3FTx4     TCTAATACACAATAAGATGGCAGGATCAGAAAAATAAATATCCAACTAAGGTTTCTATGTTTCATTTT-GCTCATCTAG-ATACTCCCTGGAATGTCATACATGCAAAGGACTATTG---------------------TGTTTTG
3FTx5     TCTAATACACAATAAGATGGCAGGATCAGAAAAATAAATATCCAACTAAGGTTTCTATGTTTCATTTT-GCTCATCTAGGATACTCCCTGGAATGTCATACATGCAAAGGACTATTG---------------------TGTTTTG
3FTx2     TCTAATACACAATAAGATGGCAGGATCAGAAAAATAAATATCCAACTAAGGTTTCTATGTTTCATTTT-GCTCATCTAGGATACTCCCTGATATGTGAAGCATGCAATCTACCAAAT---------------------TGTGATT
3FTx3     TCTAATATATAACAAGATTACAAGAATAGAAAAATAAATATCAAAGTAAGATTTCTATGTTTTAATTTCTTTCATCCAGGATACACCACAAACTGTTTCACATGTACCACTTGGACTTTGTCT---------------TGTAGAG

                                    Exon II
          <----------------------------------------------------------------------------->
   iv                                   v                              vi         vii                                       I
3FTx1     CTGTTATG---------TGTCCAGAAGGTCTTGATCAATGCTATATAAATAAAACTCTC------------TTTCCTGGTAAGCACACTCCTTATCTTTTCTCACAG-CTTTTCTTCTTGAACCCAAACTAGGGCTCCCAAATGC
3FTx4     ATCCTAAGCCA------TGTCCAGAAGGTCTTGATCAATGCTTTAAAAATGTAACTCTCTCGCTTGGTTTAATTCCTGGTAAGCACAGTCCTTATCTTTTCTCACCG-CTTTTCTTCTCAAACCCAAACTAGGGCTCCCAAATGC
3FTx5     ATCCTAAGCCA------TGTCCAGAAGGTCTTGATCAATGCTTTAAAAATGTAACTCTCTCGCTTGGTTTAATTCCTGGTAAGCACAGTCCTTATCTTTTCTCACCG-CTTTTCTTCTCAAACCCAAACTAGGGCTCCCAAATGC
3FTx2     TCCTTCCTGCTAGACCGTGTCCAAAAGGTTTTAATCAATGCTATAAAAAGTGGAATTTA---ATTGGCTTG---TCTGGTAAGCACAGTCCTTATCTTTTCTCACAGG-TTTTCTTCTTGAACCCAAACTAGAACTCCCAAATGC
3FTx3     AGTTTGAGAAA------TGTCCACCTGATAAAGGAACATGCTTTAAAAGGTGGAATTCTACT---GGCATAGCAATAAGTAAGCACAGTCCTTATCTTTTCTCACAG-CTTTTCTTCTCCAACCCAAACTAGAACTCCCAAATGC

                                                  I                                                              II
3FTx1     ACCTGGGAGTTGGAGGTCGTGTAGGGCCTGTCTGTGCTCTGCCC--------CTGAGGTCACGACCTGTGGCCAGAATATCTAAGGGGGGGGGGCAA------------------------------------------------
3FTx4     ACCTGGGAGTTGGAGGTCATGTAGGGCCTGTCTGTGCTCTGCCC--------CTAAGGTCACAACCTGTGGCCAGACAATCCAAGGGA----GGCAA------------------------------------------------
3FTx5     ACCTGGGAGTTGGAGGTCATGTAGGGCCTGTCTGTGCTCTGCCC--------CTAAGGTCACAACCTGTGGCCAGACAATCCAAGGGA----GGCAA------------------------------------------------
3FTx2     ACCTGGGAGTTGGAGGTCGTGTAGGGCCTGTATGTGCTCTCCCCGGGTTCCCCTGAGGTCACGGTCTGTAGCCAGACAA-----------GGGGCAA------------------------------------------------
3FTx3     CTTTGAGAGTTGGAGGTCCTGAAGAAGCCTTCAGTGCTCAGCCCGGGTTCCCCTGAGGTCATGGCCTGTGGCTGGACAGTCTAAGGCAGTGG-----TTCTCAAACTTTTTATTGCTGCAACCCCCGACCGGTCGTAAGTCGAGG

                                                                              II
3FTx1    --------------------------------------------------------------------------------------------------------------------------------------------------
3FTx4    --------------------------------------------------------------------------------------------------------------------------------------------------
3FTx5    --------------------------------------------------------------------------------------------------------------------------------------------------
3FTx2    --------------------------------------------------------------------------------------------------------------------------------------------------
3FTx3    ACTACTCAACTGGTCGTAAGTCGAGGACTACTCAACCGGTCGTAAGGACTACTCAACTGGTGTAGCACCATTTGCAGAATGGGGCTTTTGGCGGGCTCAGCAGGGCAGAGACAGCCTGGACAATTCCTATTGGCTTTTGAAAGCCG

                                                                              II
3FTx1     -------------------------------------------------------------------------------------------------------------------------------------------------
3FTx4     -------------------------------------------------------------------------------------------------------------------------------------------------
3FTx5     -------------------------------------------------------------------------------------------------------------------------------------------------
3FTx2     -------------------------------------------------------------------------------------------------------------------------------------------------
3FTx3     GACAGAATTAGACTCTGTTCAAGAGAGTCTCTTCTATTGGCTTTCAAAAGCCAAGAGAATAAAAGGGAGTAGCACAGCTAAGGAAAAGGGGGGGAAAAAATGGCGGACTGCGTTGTTTGGCGACCCCCGTGAAATGGTCGTTCGA
```

```
                              II                                 III                              IV
3FTx1   -------------------------------------------------------GGAAAGGGGGGCTTGGAGTGGGGAGCCCCATCTG-TCCCAGCAACAGCGTAGACTGGGGGGGAGACAATA------------ATGATGAT
3FTx4   -------------------------------------------------------GGAAAGGGGGGCTTGGAGTGGGGAGCCCCATCTG-TCCCAGCAACAGCACAGACTGGGGGGGAGACAATAATGATGGTGATGATGATGAT
3FTx5   -------------------------------------------------------GGAAAGGGGGGCTTGGAGTGGGGAGCCCCATCTG-TCCCAGCAACAGCACAGACTGGGGGGGAGACAATAATGATGGTGATGATGATGAT
3FTx2   -------------------------------------------------------GGAAAGGGGGGCTTGGAGTGGGGAGCCCCATCTG-TCCCAGCAACAGCACAGATTGGGGGAGACAA-CAACAACAACAATTAA-------
3FTx3   CCCCAAATGGGATCCCGACCCACAGGTTGAGAACCACTGGTCTAAGGAGTACAAGGGAAGAGTGGGCTTCAAATGGGGAGCCCCCTTCCATCCCAACAACAGCACAGATTGGAGGGGGGGGGC----------------------

                                                               IV
3FTx1   AATGATGATGATAATAATAAAGTTGGAAGGGACCTTGGAGGTCATCTAGTCCAATGC-------CCTGCTCAGGCAGCAAACCCTATACCATTTCGGACAAATGGCT-TCCAGTCTCTTCTTAAAAACTTCCAGTGTTGGAGCAT
3FTx4   GATGATGATGATGATGATAAGGTTGGAAGGGAACTTGGAGGTCATGTAGTCCAA-CC-------CCTGCTCAGGCAGGAAACCCTATACCATTTGAGTCAAATGGCTGTCCAGTCTCTTCTTTAAAACGTCCAGTGTTGGAGCAT
3FTx5   GATGATGATGATGATGATAAAGTTGGAAGGGAACTTGGAGGTCATGTAGTCCAA-CC-------CCTGCTCAGGCAGGAAACCCTATACCATTTGAGTCAAATGGCTGTCCAGTCTCTTCTTTAAAACGTCCAGTGTTGGAGCAT
3FTx2   AATAATAATAATAATAATAATGTTGGAAGGAACCTTGGAGGTCATCTAGTCCAATCCTCTACTGCCTGCTCAGGCAGGAC-CCCTAGACAATTTGAGACAAA----TGTCCAATCTCTTCTTAAAGACCTCCAGTGTTGGAGCAT
3FTx3   -------------------------------------------------------------------------------------------------------------------------------------------------

                                                               IV
3FTx1   TCACCTCTTCTGGAGGCAGGTG-----GTTCCCCTGGTTAATTGTTCTCTCTGTCAGGAAGTTTCTCCTTAGTTCCAGGTTGCTTCTCTCCTTGATCAG-TTCCATCCGTCGCTTCTTGTCCTGCCTTTAGGCGCCTTGGAGAAT
3FTx4   TCAGCGCTTCTGGAGGCAAGGCAAGGAGTTCCACTGGTTAATTGTTCTCACTGTCAGGAAGTTTCTCCTTAGTTCTAGGTTGCTTCTTGCCTTGGTTAATTTCCCTCCATTGCTTCTTGTCCTGCCTTCAGGCGCTTTGGAGAAT
3FTx5   TCAGCGCTTCTGGAGGCAAGGCAAGGAGTTCCACTGGTTAATTGTTCTCACTGTCAGGAAGTTTCTCCTTAGTTCTAGGTTGCTTCTTGCCTTGGTTAATTTCCCTCCATTGCTTCTTGTCCTGCCTTCAGGCGCTTTGGAGAAT
3FTx2   TCACCGCTTCTGGAGGCAGGTT-----GTTCCACTGGTTAATTGTTACAACTGTCAGGAAGTTTCTCCTTAGTTCTAGGTTGCTTCTCTCCTTGATGAGCTTCCATCCATTGCTTCTTGTCCTGCCTTCAGGCCCTTTGGAGAAT
3FTx3   -------------------------------------------------------------------------------------------------------------------------------------------------

             IV                                                                     V
3FTx1   AGCATGACTCCCTCTTCTTTAGAGAAAGGGGCGGAGGGAAAGGGGGAGGATTGGGGGTTTGTTCTGGAATTGACA--------GAAATTAGATCTCGCACATTAAATATCCAGGAGGGAGGGCATTGCCCCTGAGTCATGTCAGA
3FTx4   AGCTTGACTCCCTCTTCTTTAGAGAAAGGGGTGGAGGAAAAGGGGCAGGACTGGGGGGGTGTTGTGACATTGACA--------GAAATTAGATCTTGCACATTAAATATCCAGGAGGGAGGGCATTGCCCCCGAGTCATGTCAGA
3FTx5   AGCTTGACTCCCTCTTCTTTAGAGAAAGGGGTGGAGGAAAAGGGGCAGGACTGGGGGGGTGTTGTGACATTGACA--------GAAATTAGATCTTGCACATTAAATATCCAGGAGGGAGGGCATTGCCCCCGAGTCATGTCAGA
3FTx2   AGCTTGACTGCCTCTTCTTTAGAGAAAGGGGTGGAGGAAAAGGGGCAGGACTGGGGGTGTGTTGTGACATTGACA--------GAAATTAGATCTCGCACATTAAATATCCAGGAGGGAGGGCATTGCCCCCGAGTCATGTCAGA
3FTx3   -------------CTTCTTTAGAGAAAGGGGGAGAGAATGGCTGGGAGAGGTGTGGGTTTATTGTGACATTGGATTAAGCCATGAAATTGGATTAAGC-CATGAAATATCCAGGAGGGAGGGCATTGCCCCCGAGTCATGTCAGA

                                                               V
3FTx1   AACACAGGACTTTCCCCCAGAGAAAGGGGAAAAGTTTTCCCCTTTTTTATTTATTTACTCCTGAGTATTCCCTAGAGGAGGAAAGAGGTCTGCCTTCCCACTGGGAGGGGAATCCACCAATGTGTTAAAAGTGGGTTTACCATTT
3FTx4   AACACGGGACTTTCCCCCAGAGATAT--------------CTTTTTTTATTTATTTGCTCCTGAGTATTCCCTAGAGGAGGAAAGAGGTCCGCCTTCCCACTGNGAGGGGAATCCACCGATGTGTTAAAAGTGGCTTTACCATTT
3FTx5   AACACGGGACTTTCCCCCAGAGATAT--------------CTTTTTTTATTTATTTGCTCCTGAGTATTCCCTAGAGGAGGAAAGAGGTCCGCCTTCCCACTGGGAGGGGAATCCACCGATGTGTTAAAAGTGGCTTTACCATTT
3FTx2   AACACGGGACTTTCCCCCAGAGATAT--------------CTTTTTTTATTTATTTACTCCTGAGTATTCCCTAGAGGAGGAAAGAGGTCCGCCTTCCCACTGGGAGGGGAATCCACCGATGTGTTAAAAGTGGCTTTACCATTT
3FTx3   AACACGGGACTTTCTCCCACAGATAT--------------CTTTTTTTATTTATTTACTCCTGAGTATTCCCTAGAGGAGGAAGGAGGTCCGCCTTCCCACTGGGAGGGAAT-CCACCGATGTGTTAAAAGTGGCTTTACCATTT

                                   V                                        ↓ <------------------------ Exon III ------------------------->
                                                                                                   i                             ii      iii
3FTx1   TGTTCAGCCACAGCCCCAGATCTGTTCTTTGGCATTTTTAAAAATAATCATTCTGCTTTGTGTTTCCT-TCACAGTACTGAAGATCGAAAAGGGATGTACTACAAATTGCACTCAGACTTGGACAGAT---------AAATGTTG
3FTx4   TGTTCAGCCACAGCCCCAGATCTGTTTTTT------------AATAGTCATTCTGCTTTGTGTTTCCT-TCACAGTACTGAAGATCGAAAAGGGATGTACTACAAATTGCACTCAGACTTGGACAGAT---------AAATGTTG
3FTx5   TGTTCAGCCACAGCCCCAGATCTGTTTTTT------------AATAGTCATTCTGCTTTGTGTTTCCT-TCACAGTACTGAAGATCGAAAAGGGATGTACTACAAATTGCACTCAGACTTGGACAGAT---------AAATGTTG
3FTx2   TGTTCAGCCACAGCCCCAGATCTGTTTTTT------------AATAGTCATTCTGCTTTGTGTTTCCT-TCACAGTAATGAATATCGAAAGGGGATGTACTGCAAATTGCACTCCGAATGCGCAAACT---------AAGTGTTG
3FTx3   TGTTCAGCCACAGCCCCAGATCTGTTCCTTGGCATTTTT---AATAATTATTCTGCTTTGTGTTTCCT-TCACAGGAAGGAGGTACACCAGGGGATGTGCCGCAGCTTGCCCTAATCCGGTTGGCAATGAGAAAGTTTTCTGTTG

        <--------- Exon III --------->
                      iii
3FTx1   GCGAGACAGACAAAGTGCAACTTTATTTGA
3FTx4   GCGAGACAGACAAAGTGCAACTTTATTTGA
3FTx5   GCGAGACAAACAAAGTGCAACATTATTTGA
3FTx2   GCAAGACAAAC-CTGTGCAACGC---TTAG
3FTx3   GTGTAACAGAC-AACTGCAACAA---ATAG
```
